# Lifestyle, exercise and activity package for people living with progressive multiple sclerosis (LEAP-MS): protocol for a single-arm feasibility study

**DOI:** 10.1186/s40814-021-00852-w

**Published:** 2021-05-22

**Authors:** Julie Latchem-Hastings, Elizabeth Randell, Kate Button, Fiona Jones, Rachel Lowe, Helen Dawes, Fiona Wood, Freya Davies, Vincent Poile, Rhian O’Halloran, Barbara Stensland, Emma Tallantyre, Rebecca Playle, Adrian Edwards, Monica Busse

**Affiliations:** 1 Centre for Trials Research, Cardiff University, 4th floor Neuadd Meirionnydd, Heath Park, Cardiff, UK; 2 School of Healthcare Sciences, Cardiff University, Ty Dewi Sant, Heath Park, Cardiff, UK; 3 Faculty of Health and Social Care Science, St George’s University of London and Kingston University, London, UK; 4 Movement Science Group, Oxford Brookes University, Gipsy Lane, Headington, Oxford, UK; 5 Division of Population Medicine and PRIME Centre Wales, School of Medicine, Cardiff University, Heath Park, Cardiff, UK; 6 Helen Durham Neuro-Inflammatory Centre, University Hospital of Wales, Heath Park, Cardiff, UK

**Keywords:** Multiple sclerosis, Physiotherapy, Intervention, Physical activity, Self-management, Feasibility study

## Abstract

**Background:**

We have co-designed a tailored blended physiotherapy intervention for people with progressive multiple sclerosis (PwPMS) who often struggle to access support for physical activity. Underpinned by self-management principles, the Lifestyle, Exercise and Activity Package for people with Multiple Sclerosis (LEAP-MS) intervention incorporates face-to-face or online physiotherapy coaching sessions with an accompanying online physical activity platform. The LEAP-MS platform is a multi-user system enabling user and physiotherapist to co-create activity plans. The LEAP-MS platform consists of an information and activity suite, interactive components enabling selection of exercises into an activity programme, goal setting and activity logging. The platform also facilitates online remote support from a physiotherapist through an embedded online messaging function. We aim to evaluate the LEAP-MS platform in a feasibility trial.

**Methods:**

LEAP-MS will be evaluated within a single-arm feasibility study with embedded process evaluation. After registration and initial eligible screening, 21 participants will be required to complete baseline self-completion measures. This will be followed by an initial home-based or online coaching session with a physiotherapist (who has received tailored self-management and digital resource training) and access to the online intervention for an initial 3-month period. During this period, participants are given the option to request up to five further home-based or online physiotherapy coaching sessions. Follow-up questionnaires and semi-structured interviews will be administered 3 months after baseline with participants and intervention physiotherapists. The LEAP-MS platform will be available to participants for a further 3 months. Usage of the LEAP-MS platform will be tracked during the full 6-month period and final follow-up will be conducted 6 months after baseline.

**Discussion:**

Feasibility outcomes (recruitment, retention, intervention uptake and safety) will be reported. The process evaluation will be undertaken to identify possible mechanisms for any observed effects. The data will inform full-scale evaluations of this co-produced, blended physiotherapy intervention.

**Trial registration:**

ClinicalTrials.gov, NCT03951181. Registered 15 May 2019

**Supplementary Information:**

The online version contains supplementary material available at 10.1186/s40814-021-00852-w.

## Background

Multiple sclerosis (MS) is the most common disabling neurological disease among young adults [1] affecting an estimated 107,000 people in the UK [2]. MS is characterised by focal areas of inflammatory demyelination within the central nervous system. In the early phase of MS, most people experience discrete episodes of neurological dysfunction (relapses). However, around half of people with relapse-onset MS tend to develop an insidious deterioration in disability after 30 years from onset, which is described as secondary progressive disease [3, 4]. In addition, around 10% of people with MS do not experience relapses at onset, but rather present with an insidious accumulation of neurological disability, which continues to progress with or without superimposed relapses, termed primary progressive MS [3, 5].

In the UK, it is estimated that 10–15,000 have primary progressive MS [6] and 38,000 have secondary progressive MS [7, 8]. People with progressive MS (PwPMS) tend to have higher levels of disability than those with relapsing-remitting MS, often have high health and social care needs and self-report low health-related quality of life [9, 10]. PwPMS experience a wide range of symptoms including motor, sensory, visual, bowel and bladder dysfunction [11].

Physiotherapists play a central role as part of a multidisciplinary team of healthcare professionals who support people with progressive multiple sclerosis in the management of their symptoms [12]. Primarily focussed on maintaining, adapting or enhancing physical and sensory capabilities of individual patients, physiotherapy has been shown to be effective in, for example, improving balance [13], mobility [14] and spasticity [15] with people with MS. As part of their therapeutic toolkit, physiotherapists use exercise and have expertise in supporting and promoting physical activity [16].

Physical activity is ‘any bodily movement produced by skeletal muscles that requires energy expenditure’ [17] and relates to activities of daily living which are conducted through movement and activities with physical/movement components which are carried out as part of work, leisure or recreation—including walking or wheeling, sports, play etc. The physical activities we undertake both define and can be shaped by our lifestyle—simply defined as the way in which we live—relating to everyday behaviours and activities including work, leisure and diet [18, 19]. The interconnectedness of these concepts requires the context within which people live to be taken into consideration when exploring how to support people to be physically active.

Regular physical activity is generally regarded to be an important component of the long-term management of MS. Positive outcomes of regular physical activity include improved mobility, strength and cognition and reduced fatigue [20–22]. There are also well-established psychological and social benefits associated with physical activity in MS [23, 24], and engaging in regular physical activity is considered to be a positive way to cope with living with progressive MS [25, 26].

Engaging with physical activity significantly enough to benefit from such outcomes, however, relies on changing behaviours. Behaviour change theories (theories and models which seek to explain why people behave the way they do and what is required to alter what they do and how they think) are therefore often selected to underpin the development and testing of physical activity interventions—known as ‘behaviour change interventions’ [27].

Although physical activity interventions can be based on a plethora of behaviour change theories, aspects of social cognitive theory and self-regulation theory [28, 29] are widely drawn upon. The key constructs within these theories are those of self-efficacy and self-regulation. Self-efficacy is defined as ‘the belief in one’s capabilities to organize and execute the courses of action required to manage prospective situations’ [28]. It forms part of self-regulation, the processes of ‘self-monitoring of one’s behaviour, its determinants, and its effects; it also includes judgment in relation to personal standards and environmental circumstances; and affective self-reaction’ [29]. Methods used to implement these constructs overlap and include goal setting, feedback and guided practice.

Various physical activity interventions for people with MS have been reported in the literature, ranging from group-based to digital versatile disc (DVD) and web-based interventions [30–34]. A recent systematic review which evaluated the effectiveness of behavioural change interventions aiming to increase activity and participation in people with MS found that short duration interventions incorporating goal setting, barrier identification and information provision increased physical activity [27]. These, and more recent research exploring physical activity interventions and factors impacting physical activity levels for people with MS, highlight how developing self-determined and self-efficacious physical activity behaviours through goal setting, appropriate communication and self-monitoring are critically important determinants of sustained physical activity behaviour [35–37].

Despite the potential benefits of physical activity and the value placed on supporting people with MS to remain active, there remains little evidence about the benefits of physiotherapy or physical activity for PwPMS who have more advanced disability [38]. Most research has focussed on patients who are ambulatory, despite non-ambulatory people with MS being those who are least likely to stay active [23, 39, 40]. A systematic review [41] of physiotherapy interventions, including exercise therapy, for the rehabilitation of people with progressive multiple sclerosis published in 2016 reviewed 13 studies (of eight interventions) of variable methodological quality. It concluded that physiotherapy and exercise interventions for PwPMS were potentially of benefit but that fully powered efficacy studies were required. Recently, a home-based, self-managed standing frame programme has been found to be effective in PwPMS [42]; however, there is little research into physical activity interventions in PwPMS, which may be explained by difficulties recruiting or retaining individuals with advanced disability into research studies. These challenges, which may be explained by difficulties travelling to appointments and a high prevalence of fatigue and cognitive impairment, are the same ones that must be overcome to enable PwPMS to engage in sustained physical activity. Seeking sustainable, cost-effective interventions that facilitate access to physical activity for all remains a priority.

Despite the limited evidence about the benefits of physical activity for PwPMS, we know that people with MS, including those with progressive MS, want to keep physically active and moving [25, 43]. However, people with MS, especially those who are more disabled, find it hard to start and to maintain activity [44]. Many require support to remain physically active and often do not receive enough support [44]. When people with MS are asked about their needs, physiotherapist-led support for physical activity ranks highly [45–47] with many needing support to identify physical activity that is suited to them [48, 49]. Physiotherapists’ training and experience mean that they are ideally placed to support physical activity and exercise prescription and are often promoted as exercise experts [50]. This expert role, however, may paradoxically foster reliance on physiotherapists, and although many physiotherapists have a thorough knowledge of risk factors, pathology and their effects on all systems, they may not necessarily be confident in exercise physiology and prescription. Indeed, the first barrier to promoting activity in PwPMS may come not from the individual themselves, but from the professionals with whom they engage [51, 52].

Here we present the protocol for the LEAP-MS single-arm feasibility trial and embedded process evaluation. Underpinned by social cognitive theory and self-regulation theory, taking a self-management approach—LEAP-MS is a co-designed blended physiotherapy digital intervention [53]. Our primary objective is to establish the feasibility of the LEAP-MS intervention. Secondary to this, we will validate the underpinning intervention logic model through both qualitative assessment of intervention processes and descriptive evaluation of acceptability and patient-reported outcomes.

## Methods

### Study design summary and setting

**Fig. 1:**
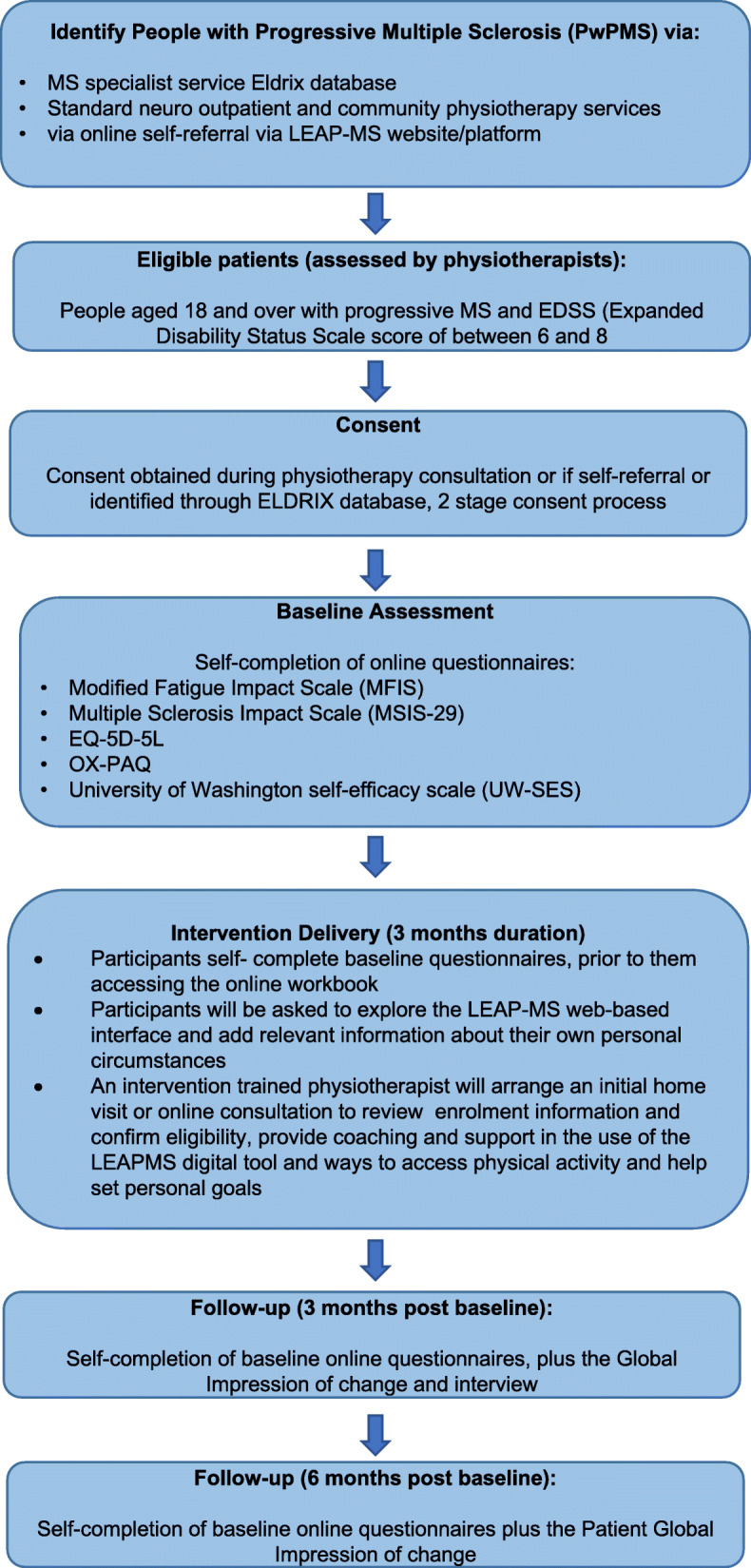
Participant flow diagram

This is a single-arm feasibility study with an embedded process evaluation. Those who are eligible and consent to participate in the study will complete a series of self-completed assessments at baseline, 3 and 6 months; during this time, they will also have access to the LEAP-MS blended physiotherapy intervention (see Fig. 1; participant flow diagram). The intervention will be delivered online and, where possible, face-face in participants’ homes. Home-based delivery is reliant upon the current COVID-19 pandemic social distancing requirements being lifted and/or local physiotherapy provision.

The intervention comprises physiotherapy coaching sessions (delivered via a secure web video conferencing system or in person where possible) and an online platform. The online platform consists of a series of co-produced resources and functions. These include online interactive education, an activity selection and planning tool, specifically developed for PwPMS and a participant-physiotherapist messaging system. The activity selection and planning tool includes tailored physical activity ideas and interactive functions enabling the development of personalised activity programmes, goal setting and activity logs. The online platform works in conjunction with remote or face-to-face coaching sessions and facilitates remote support (via the online platform messaging system) from trained physiotherapists.

#### **Population**

We will recruit 21 participants with either primary or secondary progressive multiple sclerosis (as defined by the Lublin classification) [3] who are aged 18 or over and who have an Expanded Disability Status Scale (EDSS) score [54] between 6 and 8. The EDSS scale ranges from 0 to 10 in 0.5 unit increments that represent greater levels of disability. EDSS steps 5.0 to 9.5 are defined by the impairment to walking—with people scoring 6–8 on the scale ranging from someone requiring a walking aid to walk 100m to someone who is largely restricted to a chair or wheelchair, but who maintains functional use of their arms. Participants will be required to have the capacity to consent to study participation on their own behalf and have access to mobile, wireless or wired Internet connection at home. We will exclude any individuals with relapsing-remitting or non-progressive MS, those who are unable to understand written and spoken English or those whose who are pregnant or planning a pregnancy.

The sample size is based on the 95% confidence interval for an adequate proportion of eligible subjects being recruited (70%). The lower 95% confidence interval is 50% which is the minimum acceptable recruitment proportion.

#### **Recruitment**

There will be three routes for informing potential participants about the study: (1) Eldrix HealthContact, (2) outpatient physiotherapy services or (3) MS Society branch and national MS register publicity in the local region.

1. Eldrix HealthContact is a tertiary centre MS database where PwPMS who meet the inclusion criteria and who have also given consent to be contacted about research will be identified by authorised Eldrix HealthContact users. A selection of those identified (based on EDSS scores in potentially eligible range) will be sent a study information sheet.
2. Physiotherapists at the two participating Health Boards will screen all MS outpatients for eligibility during the recruitment phase. Those eligible and interested in participating will be provided with an information sheet about the study.
3. Information about the study will be made available via the local branch of the MS Society (within the boundaries of the two participating Health Boards) and the UK MS Register inviting interested participants to complete the online expression of interest.

Those interested in participating will be required to complete an online expression of interest form and eligibility checklist via the LEAP-MS website. The expression of interest includes confirmation of MS diagnosis and EDSS self-completion. All participants recruited via the Eldrix HealthContact will have a consultant confirmed diagnosis of MS prior to entry into the database but recruitment via other routes relies on self-reporting. Having a self-enrolment option however is aimed at enhancing accessibility and inclusivity.

We will monitor population characteristics of those who express an interest in participating (age, gender and levels of disability). The expression of interest form will remain open until such time as eligibility has been confirmed for the entire recruited participant cohort. This will take the form of a two-stage process. First, prior to the initial target sample size being recruited, those who submit an expression of interest will receive an automatically generated response from the system. The message will thank them for their interest and explain that eligibility will be assessed in the order of expression of interest receipt and that the study team will contact them in due course. Second, once eligibility has been confirmed for the entire participant cohort and the study has closed to recruitment, the expression of interest page will disabled; however, interested individuals will be able to provide their contact details to receive study updates and results.

#### **Eligibility screening and informed consent**

Those who complete an online eligibility checklist and are deemed potentially eligible will receive a telephone call from the research team, to discuss what participation in the study involves and be given the opportunity to ask any questions. Their eligibility will be checked during this call and fully confirmed by their physiotherapist at the first coaching session. Those participants who are interested in the study will be directed back to the LEAP-MS website and provided with individual user details to complete an online consent form. Once the consent form has been submitted online, participants will be directed to complete the baseline assessment battery.

#### **Assessments**

All participants will be required to complete a range of patient-reported outcome measures directly online at baseline, 3 and 6 months post-baseline (plus or minus 2 weeks; see Table 1: schedule of enrolment, interventions and assessments). Selected assessments were reflective of our logic model of change, linking performance objectives to the proposed intervention outcomes over the short and medium terms [53]. In the shorter term, we anticipated that adherence to the intervention would be achieved through enhanced self-efficacy as assessed by the University of Washington 6-item short from self-efficacy scale (UW-SES-SF) (MS specific) [55]. This would then in turn influence the impact of ill-health on participation, activities and autonomy as measured by the Oxford Participation and Activities Questionnaire (OxPAQ) [56] and health-related quality of life as measured by EQ-5D-5L [57]. In the longer term, we hypothesised that the intervention would reduce the impact of the Fatigue Impact Scale (MFIS-29) [58] and the physical and psychological impact of MS from the patient’s perspective as measured by the Multiple Sclerosis Impact Scale (MSIS-29) [59]. A further consideration was participant burden and the need for self-completion measures to facilitate the remote assessment. Each of these measures has been validated for self-completion by people with multiple sclerosis.

**Table 1:** Schedule of enrolment, interventions and assessments

	Study period					
	Screening	Baseline	Intervention		Follow-up	
Timepoint	−4 weeks–0	0	0–3 months	3–6 months	3 months	6 months
**Screening**						
Pre-screening—Eldrix HealthContact Database	X					
Pre-screening—physiotherapy outpatient clinics	X					
Self-assessment online eligibility check	X					
Eligibility screen and discuss the study (telephone call from research team)	X					
Online informed consent	X					
Eligibility confirmed at physiotherapy coaching session			X			
**Intervention**						
Physiotherapy coaching sessions (up to 6 sessions)			X			
LEAP-MS activity web-based platform			X	X		
**Assessments**						
MFIS		X			X	X
MSIS-29		X			X	X
EQ-5D-5L		X			X	X
OxPAQ		X			X	X
UW-SES-SF		X			X	X
PGIC					X	X
Semi-structured interview					X	

After the baseline assessments have been completed by the participant, their online user account will be paired with an intervention physiotherapist’s account. This physiotherapist will then contact the participant to arrange the first coaching session, after which the full LEAP-MS online tool will be released to the participant.

Participants will be asked to repeat the online patient-reported outcome measures, plus a modified Patients’ Global Impression of Change (PGIC) [60], at 3 months and 6 months post-baseline. In this, they will be asked to indicate their degree of change using one of five responses (much better, slightly better, the same, slightly worse, much worse) in relation to their routine activities, emotional well-being and social engagement.

Automatic prompts will be provided to the registration email address at the start of the follow-up data collection window (2 weeks before and 2 weeks after the expected assessment completion date). Participants will receive a telephone reminder if they have not logged on in the 2 weeks prior to the expected assessment completion date. Electronic data capture will be standardised across all study remote processes using an online platform developed by the Centre for Trials Research using a bespoke Structured Query Language (SQL) database. A study-specific data management plan has been developed to ensure the security and confidentiality of all participant data and that high-quality data is available for ongoing analyses.

At the end of the initial intervention period (3 months), participants and their treating physiotherapists will be asked to participate in a semi-structured interview aimed at eliciting experiences and reflections on the intervention, and the process of its delivery (content, design, language, adaptability to personal needs and recommendations for the future). Given the small sample size of this feasibility study, everyone who consents to being interviewed will be interviewed, even if they withdraw from the intervention (see the ‘Process evaluation’ section). Interviews will be conducted by a research team member who is not involved in the intervention delivery but is a physiotherapist by background and an experienced qualitative researcher.

#### **LEAP-MS intervention**

The aim of the LEAP-MS intervention is to provide improved awareness of achievable, relevant and interesting activities and exercises for PwPMS. It will also provide an opportunity for sharing experiences of participating and enable shared management and monitoring (self and physiotherapist) of activities and exercises. It is a blended physiotherapy intervention made up of (1) a co-produced, encrypted multi-user web-based platform accessible to participants and physiotherapists and (2) coaching sessions with intervention physiotherapists delivered via a secure web video conferencing system, or in person, in the participant’s home. Intervention physiotherapists are trained on self-management principles and practice, use of technology in coaching sessions and physical activity and exercise guidelines for neurological conditions. As this is a blended intervention including a web-based platform, participants will require computer skills (or a carer companion who can assist them is needed) and Internet access for the duration of their study participation.

#### **The LEAP-MS online platform**

The platform is specifically developed for PwPMS and includes regularly updated multimedia education about being active with PMS, tailored physical activity ideas and interactive functions enabling the development of personalised activity plans, goal setting and the monitoring of activity through activity logs. Activities are displayed in an ‘activity suite’ grouped into categories, e.g. cardiovascular, strengthening, balance, flexibility, pelvic health etc., and include a large range of exercise videos from aerobics to seated boxing to tai chi. These sit alongside other, more specific exercises described or demonstrated through text and images. Each exercise or activity suggestion can be selected and added to an ‘activity plan’, enabling participants to select individual activities to try. In addition, participants are able to set and monitor personalised goals using a goal-setting function and input all activity undertaken through the use of an activity log. A fuller description and images of the intervention are described elsewhere [53].

The LEAP-MS platform enables access and data input options via desktop computers, laptops, tablets or smart phones. Participants, physiotherapists and the research team all have different access rights to and editing permissions for the LEAP-MS platform. Participants use the platform to register; complete eligibility forms, consent, baseline and follow-up measures; input safety information; and access the interactive education and activity selection and planning tool. They are also enabled to contact their intervention physiotherapist via a messaging function to ask questions, seek guidance or request coaching sessions. Physiotherapists use the platform to record coaching session notes, respond to participant questions and requests and view participant activity selections and goal setting. The platform is also used by the study team to evaluate participant engagement with the intervention and to manage data throughout the study.

#### **LEAP-MS coaching sessions**

LEAP-MS participants will meet with an intervention physiotherapist up to six times, remotely or face-face in their own homes. During coaching sessions, participants will be shown how to navigate and use the LEAP-MS online platform as required, select suitable activities, form an activity plan and set and review activity goals. Drawing on both their professional knowledge and training underpinned by the Bridges self-management approach [61], people with MS are taught how to utilise self-efficacious and self-regulatory practices throughout the consultations. Critically, physiotherapists will not instruct, or prescribe activity to participants—but instead use coaching techniques and open questioning to consider meaningful activities they would like to try and support participants to set goals, log activity and monitor their progress. Through discussion, physiotherapists will support participants to identify potential barriers (e.g. their own motivation, habits, environmental or social factors) and pre-plan ways to overcome these barriers should they arise. Following a mandatory initial coaching session, participants will be able to request up to five further coaching sessions and/or communicate via a messaging function described below.

#### **Participant use of the LEAP-MS platform and interaction with physiotherapists**

In the initial 3-month intervention period, participant use of the LEAP-MS platform works in conjunction with support from intervention-trained physiotherapists and includes a user pairing facility where patient users are paired with an intervention physiotherapist. The physiotherapist can view the activity selections and goals set by the patient participant and provide coaching and support to engage with participants in setting small targets and incorporating physical activity into their everyday life. The web-based platform also has an in-built messaging function to enable participants to contact their physiotherapist in the initial 3-month period to ask questions, seek guidance about activity engagement and request up to six coaching sessions. Coaching sessions will be conducted at participant’s homes or online dependent on participant preference whilst accommodating any local restrictions (e.g. social distancing during COVID-19 or staff availability—see [62] for COVID-19 study adaptations). When conducted online, coaching sessions will be conducted via a secure web video conferencing system. All interactions (whether in person, via web video conferencing or via the in-built platform messaging system) will operationalise a supported self-management approach to regular physical activity. Physiotherapists will not be required to respond immediately to communication through the platform, but will schedule regular time slots to respond to LEAP-MS communication, as they would with other forms of patient communication—such as returning telephone calls from outpatients or family members. They will aim to respond within 5 working days. A pre-specified inactivity period of 21 days on the website by any one participant will automatically be logged and flagged to the corresponding patient participant’s physiotherapist, who will then contact the participant to offer any further support.

Interactions between participant and physiotherapist using the in-built platform messaging function will be captured by the study database. Notes of coaching sessions will be recorded in the web platform and downloaded for adding to patient notes. Where face-to-face coaching sessions are conducted, intervention physiotherapists will be required to detail distance travelled, mode of travel time and face-to-face contact time. For coaching sessions delivered via web video conferencing, only video conferencing time will be detailed.

#### **Physiotherapist training**

All intervention physiotherapists will receive bespoke LEAP-MS training, which delivers (1) real-time training (either face-face or remotely) about self-management principles and how to integrate these into contacts with patients, (2) how to use online conferencing as a method of communication and providing consultations, (3) how to introduce/instruct patients in the use of online conferencing and online resources (in this case the LEAP-MS platform) and (4) a clinical update on physical activity and exercise guidelines for use with people with neurological conditions. The training package consists of real-time training days (delivered face-face or remotely) and an online self-study resource. The real-time self-management training workshops are delivered by experienced facilitators from Bridges Self-Management (http://www.bridgesselfmanagement.org.uk/). Paper-based learning materials traditionally accompanying face-face training workshops and video-recorded films from the training have been digitised and housed in an online learning platform for use by intervention physiotherapists to refer back to as required. Given the emergent challenges in rehabilitation service delivery during the COVID-19 pandemic, and the anticipated move to greater use of remote intervention delivery, further resources (https://www.bridgesselfmanagement.org.uk/covid-19-resources/) to help structure remote interactions were also made available as part of the final training package to ensure standardisation of coaching interactions regardless of the mode of delivery (online or face to face in the home). In addition, an accompanying online training resource included video-recordings with experienced physiotherapists with expertise in the use of digital technologies in practice to provide guidance on the use of technology in coaching sessions and videos with an expert in exercise prescription for people with neurological conditions to teach core principles of exercise physiology and prescription [63, 64]. Intervention physiotherapists are required to watch and work through these additional learning materials independently.

The physiotherapists who complete the training will be invited to participate in the LEAP-MS study as ‘intervention physiotherapists’. All those who consent to taking part will have access to conversation-based scripts that they can use to guide their coaching conversations (primarily for online use). They will also have the opportunity to practice coaching conversations and receive peer review. They will be asked to take part in interviews to share their experience of intervention training and intervention delivery.

#### **Safety**

We will assess and record any adverse events that may be reported.

##### Expected adverse events (AEs)

In this patient population, hospitalisation due to MS, acute illness resulting in hospitalisation, new medical problems and deterioration of existing medical problems are expected. This information will be self-reported by patients online and will not be subject to expedited reporting; however, it will be reviewed on a monthly basis by the study team. The physical activity intervention does not specifically involve any heavy load-bearing exercise or heavy eccentric muscle activity. However, some minor muscle soreness or muscular strain may occur in the few days following the initiation of a new exercise programme or increased physical activity. This would normally resolve spontaneously and would not require any specific interventions or additional medical care but will be noted as a potential expected related AE if reported during the 3-month intervention period. Falls and fatigue are an expected AE as part of the clinical condition but will be monitored for the duration of the intervention.

##### Procedure

Participants will be asked to use the LEAP-MS platform to self-report any incidents of falls, fatigue, increased muscle soreness or sprain, or other incidents they feel are relevant, and whether the incident required medical intervention. Selecting that medical intervention was required will trigger an automated prompt to the paired physiotherapist. Similarly, no activity on the online platform for 3 weeks will also trigger an automated prompt to the paired physiotherapist. Once prompted, the paired physiotherapist will contact the participant to discuss the incident. All serious adverse events (SAEs) that occur between the time of consent and the 3-month follow-up must be reported immediately to the Centre for Trials Research (within 24 h of knowledge of the event) by the intervention therapist using a dedicated SAE form, unless the SAE is specified as not requiring immediate reporting.

##### Planned analyses

Analyses will be guided by the CONSORT extension for pilot and feasibility studies [65]. The primary objectives are to establish feasibility of the study in terms of quantitative measures of recruitment, retention, intervention uptake and safety (see Table 2). All proportions will be tabulated with 95% confidence intervals alongside the CONSORT chart, which will detail the reasons for exclusion, refusal and dropout. Intervention uptake will be reported descriptively. There is no defined minimum dataset for the clinical secondary outcomes. Data completeness of each patient-reported outcome measure will be tabulated and will further inform our assessment of feasibility. Distributions of the outcome scores will be investigated and appropriate summary measures for the whole group tabulated with 95% confidence intervals at baseline and follow-up time points. An assessment of attrition bias will be made via tabulation of baseline characteristics for those with complete follow-up data and those who were not followed up. No formal hypothesis tests will be carried out in the analyses; however, factors such as disease severity (as represented by EDSS scores) and self-efficacy (measured by UW-SES) that may plausibly impact on adherence and retention will be explored with graphical displays. The traffic light system (green, amber, red) of progression criteria as proposed by Avery et al. [66] will be utilised (see Table 2) to guide our decisions as to future evaluations.

**Table 2:** Feasibility outcomes

Feasibility outcome	Measurement	Green	Amber	Red
**Recruitment**	Percentage of those submitting online permission to contact forms who are eligible and who consent to participation	70%	50–69%	Less than 50%
**Retention**	Percentage of individuals who complete the 3-month follow-up assessments	70%	50–69%	Less than 50%

If at the end of the study the feasibility progression criteria are achieved, then the recommendation would be to move to a randomised evaluation. Modifications in the trial processes or the intervention may be required if progression criteria are not fully achieved. If there is not an identifiable reason or remediable action that can be taken, then progression to a full trial would not be recommended.

Intervention uptake and safety are not formal progression criteria in this single-arm feasibility study but will be closely monitored and considered in any final recommendations for further evaluations. Intervention uptake will be reflected by (1) the percentage of initial coaching sessions completed, the number of additional physiotherapy coaching sessions requested and completed and the number of remote physiotherapist contacts recorded and (2) frequency and duration of weekly logged physical activity. Website log in rates and length of time between each log in episode will provide supplemental information on intervention uptake. Safety will be assessed using an online process of self-reporting by the participant and from any SAE forms completed by the intervention therapist.

### Process evaluation

The process evaluation will enable an understanding of the acceptability and fidelity of the intervention, identify possible mechanisms for any observed effects and learn about any adaptations made by the participants (PwPMS and physiotherapists) in undertaking the programme. Acceptability assessment will focus on content, design, language and adaptability to personal needs. It will be assessed through the analysis of any remote contact between participants and physiotherapists as well as semi-structured interviews (conducted either face to face or via the secure web video conferencing system) with participants who completed the intervention, those who withdraw from the study and physiotherapists who deliver the intervention. In physiotherapist interviews, we will collect detailed demographic information so we can understand how their characteristics (work setting, previous experience, previous training) influence their approach, experience of the training and delivering the intervention.

Fidelity of the LEAP-MS intervention delivery will be assessed using independent analysis of audio-recorded and/or observed (sampled) sessions, analysis of participants’ online activity logs and participant-physiotherapist communications. Six initial coaching sessions (approximately 25% of initial coaching sessions) and between 5 and 10 follow-up coaching sessions (dependent on participant consent) will be observed and audio-recorded if conducted face-face. Coaching sessions conducted via web video conferencing will be recorded using an in-built recording function.

Actions observed during face-face observations will be captured using basic proxemic sketches (stick people drawings) and kinesics (kinesics refers to the nonverbal movement-related elements of communication in the creation and sustaining of social interactions), alongside standard ethnographic notations (the description of actions/happenings as the observer sees them) [67]. The proxemic and kinesics sketches will serve to record the physical and spatial interactions between patient, physiotherapist and intervention technology, to assist the charting of how learning is delivered and ‘gets done’ through the coaching sessions. If the intervention is delivered remotely, coaching sessions will be recorded and analysed directly.

This range of data collection methods has been selected to enable a comprehensive and multi-faceted description of the experience of those taking part, a nuanced understanding of intervention delivery and usage. The multiple methods selected will also act as a robust form of triangulation.

All qualitative data collection for the process evaluation will be carried out by a research team member with qualitative research experience, not involved in intervention delivery. All data will initially be separated into their ‘type’, i.e. speech, text or action (observation), with appropriate methods of analysis applied to each type of data. Interviews, recorded coaching sessions and observations will be analysed thematically initially [68]. Should the coaching sessions component of the intervention be found to be central to the usage of the online platform, discourse analysis may be conducted to better capture and understand the impact of interaction and communication between patient participant and physiotherapist. Data collected from text-based online interaction (emails between patient participant and physiotherapist, goal setting and activity records) will be considered as ‘personal documents’ and subject to textual analysis methods.

Due to the focus of this process evaluation on the use of each component of the intervention, the personal documentation will be initially subjected to content analysis—with a more detailed thematic analysis applied if required [69], to assist final synthesis and triangulation should the depth of the data warrant it. Key findings from each type of data set will be compared and contrasted, drawing out similarities, differences and any contradictions. Data will be reviewed in light of any contradictions and will guide a member checking process with participants, which will be conducted prior to the write-up and dissemination of findings.

**Fig. 2:**
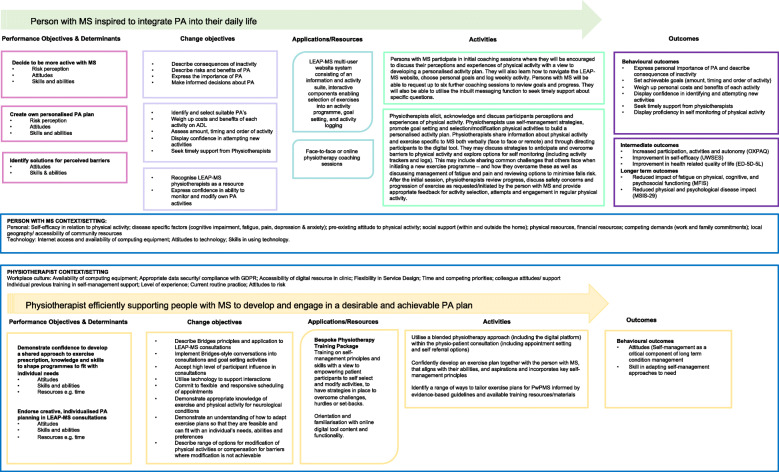
The LEAP-MS intervention logic model detailing objectives, activities and outcomes of PwPMS and the intervention physiotherapist

Findings will be separated into process and outcome data ready for reporting but with consideration being given to where/if the doing of any process is a major contributor to the outcomes or perceived experience of participants. At this point, we will explore possible mechanisms for any observed effects (both for the person with MS and the intervention physiotherapist) so as to validate change objectives, behavioural outcomes and patient-reported intermediate and longer-term outcomes as depicted in the proposed intervention logic model (Fig. 2). There are training needs for physiotherapy staff in delivering this intervention. These are reflected in the physiotherapy component of the logic model as are aspects related to the broader context within which this intervention will be implemented.

## Discussion

The LEAP-MS platform is a multi-user system enabling participants and physiotherapist to co-create activity plans. The LEAP-MS platform consists of an information and activity suite, interactive components enabling selection of exercises to create an activity programme, goal setting and activity logging. The platform also facilitates remote support from a physiotherapist through an embedded online messaging function. Our experience here lays the basis for the development of multi-user platforms that can be adapted according to population and trial design.

As the secure, encrypted multi-user web-based platform will be accessible to participants, physiotherapists and researchers with data input options via desktop computers, laptops, tablets or smart phones, participants are able to use the platform to register; complete eligibility forms, consent, baseline and follow-up measures; and access the intervention. Participants, physiotherapists and research administrators all have different access and editing level permissions within the platform. It is also used by the study team to evaluate participant engagement with the intervention and to manage data throughout the study.

Our ambition has been to co-design a model for physical activity self-management support for PwPMS that is patient-, family/carer- and community-centred with physiotherapists providing a unique role as a coach and partner throughout the whole disease trajectory [70]. Self-management approaches are associated with a reduced reliance on health professionals and an increased sense of autonomy and control over an individual’s condition. Such approaches are also characterised by upskilling the individual to anticipate potential barriers to achieving any specified goals and to problem solve in the face of such challenges. Any programme, based on self-management principles, should then, at its heart, address the fundamental, individual and relational barriers that typical physio-led interventions may pose. Upskilling physiotherapists’ self-management support skills alongside exercise prescription knowledge, sharing expertise and working collaboratively with people living with progressive MS to define strategies and activity plans is more likely to promote physical activity behaviour change [71].

Unlike other physiotherapy-based online activity platforms for other conditions or general education platforms, the LEAP-MS platform has a paired account function in which people with MS can be paired with their physiotherapist. Critically, rather than the physiotherapist selecting and prescribing activities, the person with MS has complete choice and control of this process. The physiotherapist can view participant activity logs, but advise only as required by the person with MS. Furthermore, the patient-facing element of the LEAP-MS intervention platform combines multimedia educational content, activity provision, activity monitoring and goal setting. It includes an online hub for physiotherapists, which draws together self-management training and provides a space for multimedia exercise in long-term neurological conditions.

Evaluation of feasibility, including intervention uptake as measured by login rates and duration, and acceptability in terms of content, design, language and adaptability to personal needs will inform modification and future evaluation. Findings from the feasibility study will be disseminated to participants, healthcare professionals and the public via a series of outputs. These include a lay summary of findings to be sent to participants and published on university and funder websites for public viewing, formal research reports, peer-reviewed publications and conference papers to share findings with healthcare professionals.

### Trial status

The trial is sponsored by Cardiff University (resgov@cardiff.ac.uk) and is set up. Recruitment will commence on 01.06.2020 and is anticipated to end on 30.10.2020. This manuscript has been drafted according to version 1.1 (12/05/2020) of the trial protocol. The protocol has been written according to the Standard Protocol Items: Recommendations for Interventional Trials (SPIRIT) statement (see Fig. 2 and Additional File 1); the intervention is described according to the Template for Intervention Description and Replication (TIDieR) checklist (see Additional File 2); and the final report will follow the Consolidated Standards of Reporting Trials (CONSORT) statement (Extension for Pilot and Feasibility Studies). Study results will be published on ClinicalTrials.gov and in peer-reviewed literature.

## Supplementary Information

**Additional file 1:.** SPIRIT 2013 Checklist

**Additional file 2:.** The TIDieR (Template for Intervention Description and Replication) Checklist

## Data Availability

We aim to make our research data available wherever possible, subject to regulatory approvals, any terms and conditions placed upon us from external providers, patient confidentiality and all laws concerning the protection of personal information.

## Abbreviations

LEAP-MS: Lifestyle, Exercise and Activity Package for people with Multiple Sclerosis
MS: Multiple sclerosis
PwPMS: People with progressive MS
DVD: Digital versatile disc
EDSS: Expanded Disability Status Scale
MFIS: Modified form of the Fatigue Impact Scale
MSIS-29: Multiple Sclerosis Impact Scale
EQ-5D-5L: A health-related quality of life outcome measure
OxPAQ: Oxford Participation and Activities Questionnaire
UW-SES: University of Washington 6-item short form self-efficacy scale
PGIC: Patients’ Global Impression of Change
AE: Adverse event
SAE: Serious adverse event
NHS: National Health Service
SPIRIT: Standard Protocol Items: Recommendations for Interventional Trials
TIDieR: Template for Intervention Description and Replication
CONSORT: Consolidated Standards of Reporting Trials
